# Pregnancy and birth characteristics of Aboriginal twins in two Australian states: a data linkage study

**DOI:** 10.1186/s12884-021-03945-9

**Published:** 2021-06-28

**Authors:** Alison J. Gibberd, Jessica Tyler, Kathleen Falster, David B. Preen, Mark Hanly, Marilyn J. Clarke, Bridgette J. McNamara, Sandra J. Eades, Katrina J. Scurrah

**Affiliations:** 1 Melbourne School of Population and Global Health, The University of Melbourne, Parkville, Australia; 2 School of Population Health, The University of New South Wales, Sydney, Australia; 3 School of Population and Global Health, The University of Western Australia, Crawley, Australia; 4 Centre for Big Data Research in Health, The University of New South Wales, Sydney, Australia; 5 Mid North Coast Local Health District, Port Macquarie, Australia; 6 Curtin Medical School, Curtin University, Perth, Australia

**Keywords:** Indigenous, Aboriginal and Torres Strait Islander, Twins, Multiples, Pregnancy, Birth, Perinatal, Linked data

## Abstract

**Introduction:**

Perinatal outcomes for singleton pregnancies are poorer, on average, for Aboriginal people than non-Aboriginal people, but little is known about Aboriginal multifetal pregnancies. Yet multifetal pregnancies and births are often more complicated and have poorer outcomes than singleton pregnancies. We describe the pregnancies, births and perinatal outcomes for Aboriginal twins born in Western Australia (WA) and New South Wales (NSW) with comparisons to Aboriginal singletons in both states and to non-Aboriginal births in NSW.

**Materials and methods:**

Whole-population birth records and birth and death registrations were linked for all births during 2000–2013 (WA) and 2002–2008 (NSW). Hospital records and the WA Register of Developmental Anomalies - Cerebral Palsy were linked for all WA births and hospital records for a subset of NSW births. Descriptive statistics are reported for maternal and child demographics, maternal health, pregnancy complications, births and perinatal outcomes.

**Results:**

Thirty-four thousand one hundred twenty-seven WA Aboriginal, 32,352 NSW Aboriginal and 601,233 NSW non-Aboriginal births were included. Pregnancy complications were more common among mothers of Aboriginal twins than Aboriginal singletons (e.g. 17% of mothers of WA twins had hypertension/pre-eclampsia/eclampsia vs 8% of mothers of singletons) but similar to mothers of NSW non-Aboriginal twins. Most Aboriginal twins were born in a principal referral, women’s or large public hospital. The hospitals were often far from the mother’s home (e.g. 31% of mothers of WA Aboriginal twins gave birth at hospitals located more than 3 h by road from their home). Outcomes were worse for Aboriginal liveborn twins than Aboriginal singletons and non-Aboriginal twins (e.g. 58% of NSW Aboriginal twins were preterm compared to 9% of Aboriginal singletons and 49% non-Aboriginal twins).

**Conclusions:**

Mothers of Aboriginal twins faced significant challenges during the pregnancy, birth and the postnatal period in hospital and, in addition to accessible specialist medical care, these mothers may need extra practical and psychosocial support throughout their journey.

**Supplementary Information:**

The online version contains supplementary material available at 10.1186/s12884-021-03945-9.

## Introduction

While the majority of Aboriginal and Torres Strait Islander (hereafter respectfully called Aboriginal) mothers and babies have positive pregnancy and birth outcomes, national data continue to show disproportionate adverse outcomes for Aboriginal compared to non-Aboriginal Australians [1]. Aboriginal infants have almost twice the rate of low birth weight, preterm birth and perinatal mortality as non-Aboriginal infants [1]. Poor perinatal health is associated with subsequent development and health [2–4]. To drive improvements, the Coalition of Aboriginal and Torres Strait Islander Peak Organisations and Australian state and federal governments recently set a new Closing the Gap outcome – “Children are born healthy and strong” – with a target of 91% of Aboriginal liveborn singletons having a healthy birth weight by 2031 [5].

Notably, this new Closing the Gap target excludes multiple births. However, Aboriginal twins and higher multiples may be a particularly high-risk group. Studies in non-Aboriginal populations report that serious complications are more common in multifetal pregnancies than singletons, including anaemia, pre-eclampsia and possibly gestational diabetes, and complications such as twin-to-twin transfusion syndrome are unique to multiple pregnancies with monochorionicity [6, 7]. Multiples are also much more likely than singletons to have adverse birth outcomes such as preterm birth, low birth weight, perinatal mortality and postnatal morbidity [8–10]. Specifically, perinatal mortality rates are approximately 3-times higher in twins and 12-times higher in higher-order multiples than singletons [1]. Given these differences, guidelines recommend heightened monitoring of multifetal pregnancies and access to specialist medical care during birth [11, 12].

Multiples account for approximately 3% of babies born each year in Australia [1] yet there is little epidemiological information available on Aboriginal multifetal pregnancies and births. Australia’s annual report on pregnancies and births does not separately report on Aboriginal multiples and researchers frequently exclude multiples from analyses [1, 13]. Knowledge of these pregnancies and births is needed to inform the health service response to a population group at high risk of experiencing adverse outcomes. Many Aboriginal mothers live in remote and low socioeconomic status areas, far from the hospitals that provide specialist obstetric and infant care, including neonatal intensive care units (NICUs). For example, all NICUs in the state of Western Australia (WA) are located in Perth, more than 1500 km from the state’s northern-most region, the Kimberley, where 22% of Aboriginal mothers live (and 1% of non-Aboriginal mothers) [14]. Furthermore, delayed and less frequent antenatal care is more common among women who experience socioeconomic deprivation or live in remote areas [8].

This paper aims to fill the gap in knowledge of Aboriginal twins by describing the pregnancies, births and perinatal outcomes for all Aboriginal twins born in WA from 2000 to 2013 and New South Wales (NSW) from 2002 to 2008 using linked administrative data, alongside data on Aboriginal singletons in both states and non-Aboriginal births in NSW.

## Materials and methods

### Data sources

Birth records from the Midwives Notification System (WA) and Perinatal Data Collection (NSW), birth registrations, inpatient hospital records from the Hospital Morbidity Data Collection (WA) and Admitted Patients Data Collection (NSW), WA Register of Developmental Anomalies - Cerebral Palsy (WARDA), and death registrations were linked probabilistically by the Data Linkage Branch (WA) and the Centre for Health Record Linkage (NSW). Birth records in both states include deliveries of all live-born or stillborn infants following at least 20 weeks’ gestation. Some information could not be reported for both states because it was collected by one state only.

### Study population

WA mothers and babies, 2000–2013, Aboriginal only: The WA study population was all Aboriginal births in WA from 2000 to 2013 (*N* = 34,127), following exclusion of one infant recorded as a twin who did not link to a corresponding twin. This cohort’s data were obtained for the Defying the Odds project [15]. Infants were classified as Aboriginal if they or a parent or grandparent were listed as Aboriginal on their Indigenous Status Flag. This flag assigns Aboriginal status based on a range of administrative records and strikes a balance between under-ascertainment of Aboriginal people and incorrect categorisation of non-Aboriginal people as Aboriginal [16, 17]. Further details regarding the algorithm used to create this flag variable are available in Christensen et al. [18]

NSW mothers and babies, 2000–2008, Aboriginal and non-Aboriginal: The NSW study population included all Aboriginal (*N* = 32,352) and non-Aboriginal (*N* = 601,233) births in NSW from 2002 to 2008, after exclusion of 23 infants recorded as multiples who did not link to corresponding multiples. Additional file 1 provides information about the linkage. Children were classified as Aboriginal if their birth record listed their mother as Aboriginal or their birth registration recorded either parent as Aboriginal. Hospital data were only available for 164,727 (26%) children (164,588 singletons and twins) who started school and had an Australian Early Development Census (AEDC) record in 2009 or 2012 (known as the Seeding Success cohort) [19]. Variables derived using hospital data are reported in Additional file 2 for the subset of children who started school in 2009/2012.

The datasets in this study include many Aboriginal communities in Australia; 45% of all Aboriginal Australians live in the states of WA and NSW [20].

### Analysis variables

Demographic characteristics: We report maternal age, parity, prior multiple birth (WA only), and private hospital insurance when admitted to hospital for the birth. Quintiles of socio-economic disadvantage were formed using the Australian Bureau of Statistics’ Socio-Economic Indexes for Areas Index of Relative Socio-Economic Disadvantage [21]. Further details of disadvantage are in Additional file 1. The remoteness of the mother’s residence at the time of the birth was assigned according to the Accessibility/Remoteness Index of Australia Plus (ARIA+) categories (major cities, inner regional, outer regional, remote and very remote) [22].

Maternal health and pregnancy complications: We report whether the mother’s first antenatal visit was at or before 14 weeks’ completed gestation, after 14 weeks or no antenatal visits. In WA, this information was only available from 2010 onwards. Maternal health conditions included gestational diabetes, pre-existing diabetes, pre-eclampsia/eclampsia/gestational hypertension, and pre-existing hypertension, obtained from the infant’s birth record. Additionally, for WA and the NSW 2009/2012 school starters, we also used the mother’s birthing admission to obtain diagnoses, as using both birth and hospital records improves classification [23]. Complications of the pregnancy included threatened abortion (< 20 weeks’ gestation), antepartum haemorrhage, threatened preterm labour, and preterm prelabour rupture of membranes. In addition to the birth record, these complications were ascertained from maternal hospital admissions during the pregnancy and birth. The International Statistical Classification of Diseases, Tenth Revision, Australian Modification (ICD-10-AM) [24] codes for these conditions are shown in Additional file 3.

Birth: The hospital where the mother gave birth and transfers from another hospital prior to the birth were identified from hospital records in WA and birth records in NSW. The birth hospital was categorised using peer groups defined by the Australian Institute of Health and Welfare (public principal referral/women’s [peer groups D1 and D10], large public [peer groups D2 and D10], small public [remaining public hospitals], private, and born outside hospital) [25]. In both NSW and WA birth records, the small number of births outside hospital are flagged. Estimated travel times by road from the mother’s home to the birth hospital are reported. In NSW, births outside hospital with an independent midwife were identified and travel times were set to zero minutes. In WA, home births could not be distinguished from other births outside hospital and travel times were not estimated. Methodological details of the estimation of travel times are outlined in Additional file 1.

Other birth details reported are presentation (vertex, breech and other), labour (spontaneous, induced and no labour), use of instruments and whether the birth was vaginal or caesarean. For WA, we further distinguished between planned and emergency caesareans. WA birth records list whether the birth was attended by an obstetrician, other medical practitioner, midwife, student and/or no-one/other. NSW birth records list categories of care for the birth as private obstetrician, hospital-based medical, general practitioner, hospital-based midwife, and/or independent midwife.

Perinatal outcomes: These included stillbirth (fetal death after at least 20 completed weeks’ gestation), neonatal death (death less than 28 days after a live birth), gestational age, and birth weight. Outcomes of live births included resuscitation and admission to a NICU/special care nursery (SCN). Length of hospital stay was available for WA and the NSW 2009/2012 school starters and was defined as (a) days from birth to final hospital discharge or (b) for WA ‘healthy liveborn’ infants with no hospital birth admission record, length of stay listed on the birth record. In WA, infants classified as ‘healthy liveborn’ infants did not have a birth hospital admission available (57% of the cohort). ‘Healthy liveborn’ infants had an ICD-10-AM principal diagnosis code commencing with “Z38”.

Cerebral palsy diagnosed by 6 years of age and recorded in the WARDA was also reported. Maternal length of hospital stay was calculated as the number of days from her first admission to her final hospital discharge, including transfers to other (non-psychiatric) hospitals. Postpartum haemorrhage (PPH) requiring blood transfusion has been comprehensively listed in NSW birth records since 2007. In WA, a PPH requiring blood transfusion was indicated if (1) the mother’s birthing admission or her child’s birth record had a PPH diagnosis and (b) her birthing admission listed a blood transfusion (ICD-10-AM codes are in Additional file 3).

### Statistical analyses

Counts and proportions are reported for categorical variables. Means and standard deviations or medians and interquartile ranges were calculated for continuous variables. Comparisons were made between: Aboriginal twins and Aboriginal singletons in WA and NSW, non-Aboriginal twins and non- Aboriginal singletons in NSW, and Aboriginal twins and non-Aboriginal twins in NSW. Pearson’s chi-squared test was used to compare proportions for all variables, except remoteness, socioeconomic disadvantage, and maternal and infant length of hospital stay [26]. The Cochran-Armitage test for trend was used for remoteness and socioeconomic disadvantage [27]. Comparisons of length of hospital stay were made using Wilcoxon rank sum tests [26]. The tables include indicators of whether *P*-value< 0.001 and *P*-value< 0.05.

## Results

The WA cohort consisted of 33,229 Aboriginal singletons, 880 twins and 18 triplets. In NSW, there were 31,524 Aboriginal singletons, 794 twins, 30 triplets and 4 quadruplets and 582,355 non-Aboriginal singletons, 18,338 twins, 516 triplets and 24 quadruplets. Multiples made up 2.6% of the Aboriginal babies in WA and NSW and 3.1% of the non-Aboriginal babies in NSW.

### Demographic characteristics

Mothers of twins were at least 1 year older, on average, than mothers of singletons for each of the three groups: Aboriginal WA births, Aboriginal NSW births and non-Aboriginal NSW births (Table 1). For Aboriginal births, nulliparity was less common among mothers of twins than mothers of singletons (WA twins: 27% nulliparous, WA singletons: 32%, NSW twins: 28%, NSW singletons: 35%), but for non-Aboriginal NSW infants, nulliparity was similar among mothers of twins and singletons (twins: 44%, singletons: 42%). Compared to mothers of singletons, the proportions of mothers of twins living in the most advantaged areas and major cities were 1 to 3 percentage points higher for both Aboriginal and non-Aboriginal births.

**Table 1:** Demographics of Western Australian (2000–2013) and New South Wales’ (2002–2008) births by plurality and Aboriginality

	Western Australia (2000–2013)		New South Wales (2002–2008)			
	Aboriginal		Aboriginal		Non-Aboriginal	
Demographics	Twin ***N*** = 880 n (%)	Singleton ***N*** = 33,229 n (%)	Twin ***N*** = 794 n (%)	Singleton ***N*** = 31,524 n (%)	Twin ***N*** = 18,338 n (%)	Singleton ***N*** = 582,355 n (%)
Groups compared and statistical significance reported^1^	A	A	B	B	C	C
			D		D	
**Maternal characteristics**						
Age (mean (SD))	26.6 (5.9)	24.9 (6.0)	27.5 (6.2)	25.7 (6.2)	31.5 (5.2)	30.1 (5.5)
Age group ^ABCD^						
< 20	112 (13)	6909 (21)	75 (9)	5616 (18)	268 (1)	17,969 (3)
20–24	232 (26)	10,561 (32)	217 (27)	9537 (30)	1610 (9)	79,062 (14)
25–29	282 (32)	8165 (25)	208 (26)	7662 (24)	4256 (23)	160,857 (28)
30–34	156 (18)	5026 (15)	164 (21)	5609 (18)	6893 (38)^2^	198,560 (34)
35+	98 (11)	2560 (8)	130 (16)	3082 (10)	5307 (29)^2^	125,749 (22)
Parity (mean (SD))	1.9 (1.9)	1.7 (1.8)	1.8 (1.8)	1.5 (1.7)	0.9 (1.2)	1.0 (1.1)
Parity group ^ABCD^						
0	234 (27)	10,773 (32)	224 (28)	11,058 (35)	8120 (44)	244,868 (42)
1	220 (25)	8352 (25)	206 (26)	8370 (27)	6003 (33)^2^	197,772 (34)
2+	426 (48)	14,104 (42)	364 (46)	12,024 (38)	4181 (23)^2^	138,629 (24)
Previous multiple birth^A^	26 (3)	465 (1)	–	–	–	–
Insurance at time of birth^a^	64 (7)	1621 (5)	–	–	–	–
Socioeconomic disadvantage^BCD^						
Quintile 1 (most advantaged)	64 (7)	1946 (6)	56 (7)	1246 (4)	4489 (25)	125,066 (22)
Quintile 2	82 (9)	3083 (9)	74 (9)	2877 (9)	3059 (17)	93,835 (16)
Quintile 3	156 (18)	5249 (16)	166 (21)	5906 (19)	3639 (20)^2^	115,981 (20)
Quintile 4	182 (21)	7612 (23)	278 (35)	12,240 (39)	4115 (23)^2^	138,903 (24)
Quintile 5 (most disadvantaged)	390 (45)	14,778 (45)	214 (27)	8982 (29)	2884 (16)	103,054 (18)
Remoteness of residence^abCD^						
Major cities	394 (45)	14,004 (42)	386 (49)	14,410 (46)	14,702 (81)	456,767 (79)
Inner regional	66 (8)	2131 (6)	256 (32)	10,052 (32)	2671 (15)^2^	91,304 (16)
Outer regional	128 (15)	5272 (16)	120 (15)	5436 (17)	761 (4)2	26,874 (5)
Remote	168 (19)	6187 (19)	20 (3)	1217 (4)	52 (< 1)	1806 (< 1)
Very remote	124 (14)	5585 (17)	6 (1)	136 (< 1)	0 (0)	88 (< 1)
**Infant characteristics**						
Sex^C^						
Male	450 (51)	16,823 (51)	404 (51)	16,169 (51)	9190 (50)	299,820 (51)
Female	430 (49)	16,404 (49)	390 (49)	15,350 (49)	9146 (50)	282,499 (49)
Same sex twin^3^	648 (74)	–	546 (69)	–	12,252 (67)	–

*SD* standard deviation. ^1^The superscripts ^ABCDabcd^ which appear next to the demographic factors indicate whether two groups were statistically significantly different: ^A^ or ^a^ for a comparison of WA Aboriginal singletons and twins; ^B^ or ^b^ for a comparison of NSW Aboriginal singletons and twins; ^C^ or ^c^ for a comparison of NSW non-Aboriginal singletons and twins; and ^D^ or ^d^ for a comparison of NSW Aboriginal twins and NSW non-Aboriginal twins. Pearson’s chi-squared tests were conducted for binary variables and Cochran-Armitage trend test for ordinal variables. A capital letter indicates *p* < 0.001 and a lower-case letter indicates 0.001 < *p* < 0.05. ^2^Some twin pairs had inconsistent information on their two birth records. ^3^Data were not available on zygosity. Data were missing for: maternal age (0 infants in WA, 180 infants in NSW), maternal parity (0 infants in WA, 1192 infants in NSW), insurance at time of birth (380 infants in WA), socioeconomic disadvantage (567 infants in WA, 5947 infants in NSW), remoteness of residence (50 infants in WA, 5947 infants in NSW), sex (2 infants in WA, 43 infants in NSW) and same sex twin (4 infants - 2 twin pairs - in WA)

Comparing mothers of Aboriginal twins and non-Aboriginal twins, mothers of Aboriginal twins from both states were at least 4 years younger than mothers of non-Aboriginal twins from NSW and they resided in more remote and disadvantaged areas.

### Maternal health and pregnancy complications

Antenatal care in the first trimester was more common for twin pregnancies than singleton pregnancies for both Aboriginal and non-Aboriginal pregnancies (Table 2). The prevalence of pre-existing conditions was similar for mothers of twins and singletons. Gestational diabetes was also similar at 5% for mothers of WA Aboriginal singletons and twins, 4% for NSW Aboriginal singletons and twins, and 5% for NSW non-Aboriginal singletons and 6% for non-Aboriginal twins. When both hospital and birth records were used to ascertain gestational diabetes among the NSW 2009/2012 school starters, the proportion of women diagnosed with gestational diabetes was two percentage points higher for twin pregnancies compared to singleton pregnancies (Aboriginal twins: 6%, Aboriginal singletons: 4%, non-Aboriginal twins: 8%, non-Aboriginal singletons: 6%) (Additional file 2, Table S1). Other pregnancy complications were more common among mothers of twins compared to singletons; for example, in WA, pre-eclampsia/eclampsia/gestational hypertension were twice as common in mothers of Aboriginal twins (17%) compared to mothers of Aboriginal singletons (8%). These hypertensive disorders were less common in the mothers of NSW babies when only the Perinatal Data Collection diagnoses were considered (Table 1), but similar to the WA cohort when hospital diagnoses were also included for the subset of children who started school in 2009/2012 (Additional file 2, Table S1).

**Table 2:** Pregnancies in Western Australia (2000–2013) and New South Wales (2002–2008) by plurality and Aboriginality

	Western Australia (2000–2013)		New South Wales (2002–2008)			
	Aboriginal		Aboriginal		Non-Aboriginal	
Characteristic	Twin ***N*** = 880 n (%)	Singleton ***N*** = 33,229 n (%)	Twin ***N*** = 794 n (%)	Singleton ***N*** = 31,524 n (%)	Twin ***N*** 18,338 n (%)	Singleton ***N*** = 582,355 n (%)
Groups compared and statistical significance reported^1^	A	A	B	B	C	C
			D		D	
Gestation at first antenatal visit^bCD, 2^						
13 weeks or less	120 (49)	3797 (45)	508 (64)	18,955 (60)	13,894 (76)	410,156 (70)
14 weeks or more	n.p.	4551 (54)	270 (34)	11,545 (37)	4244 (23)	166,711 (29)
No visit (WA) / No visit or missing (NSW)	n.p.	125 (1)	16 (2)	1024 (3)	200 (1)	5488 (1)
Maternal health and pregnancy complications						
Gestational diabetes^Cd^	48 (5)	1724 (5)	28 (4)	1105 e(4)	1095 (6)^3^	26,541 (5)
Pre-existing diabetes^bCd^	8 (1)	557 (2)	12 (2)	230 (1)	142 (1)	3315 (1)
Pre-eclampsia/eclampsia/gestational hypertension^ABCd^	148 (17)	2650 (8)	69 (9)	1618 (5)	2087 (11)^3^	31,855 (5)
Pre-existing hypertension^C^	18 (2)	415 (1)	8 (1)	286 (1)	237 (1)^3^	5547 (1)
Threatened abortion^A^	56 (6)	1002 (3)	–	–	–	–
Antepartum haemorrhage^A^	84 (10)	1787 (5)	–	–	–	–
Threatened preterm labour^A^	256 (29)	3099 (9)	–	–	–	–
Preterm prelabour rupture of membranes^A^	174 (20)	1695 (5)	–	–	–	–

*n.p.* counts not publishable because of privacy concerns as they are either less than 5 or could lead to calculation of a count of less than 5. ^1^The superscripts ^ABCDabcd^ which appear next to the demographic factors indicate whether two groups were statistically significantly different: ^A^ or ^a^ for a comparison of WA Aboriginal singletons and twins; ^B^ or ^b^ for a comparison of NSW Aboriginal singletons and twins; ^C^ or ^c^ for a comparison of NSW non-Aboriginal singletons and twins; and ^D^ or ^d^ for a comparison of NSW Aboriginal twins and NSW non-Aboriginal twins. Pearson’s chi-squared tests were conducted. A capital letter indicates *p* < 0.001 and a lower-case letter indicates 0.001 < *p* < 0.05. ^2^WA and NSW have different definitions of gestational age at first antenatal visit. In WA, this information is reported by birth hospital and information on antenatal visits outside the hospital may be unavailable. Gestation at first antenatal visit was not recorded in WA until 2010 and data were missing for 25,410 infants in WA. This information was collected in NSW throughout the study period but from 2002 to 2005 mothers who did not receive any antenatal care could not be distinguished from mothers who did receive antenatal care, but their data was missing, so the two groups were combined in NSW. ^3^Some twin pairs had inconsistent information on their two birth records

Comparing NSW Aboriginal and non-Aboriginal twin pregnancies, the likelihood of pregnancy complications was similar, with the exception of antepartum haemorrhage among the NSW 2009/2012 school starters (13 and 6% for Aboriginal and non-Aboriginal twin pregnancies, respectively, and 4 and 3% for Aboriginal and non-Aboriginal singleton pregnancies).

### Birth

The majority of WA Aboriginal twins were born in public principal referral/women’s hospitals (65%) compared to only 26% of singletons (Table 3). In NSW, the most common hospital category for the birth of Aboriginal twins was large hospital (50%), followed by public principal referral/women’s hospital (41%). For non-Aboriginal twins, the most common hospital category was public principal referral/women’s hospital (47%), followed by private hospital (25%).

**Table 3:** Birth characteristics in Western Australia (2000–2013) and New South Wales (2002–2008) by plurality and Aboriginality

	Western Australia (2000–2013)		New South Wales (2002–2008)				
	Aboriginal		Aboriginal		Non-Aboriginal		
Characteristic	Twin ***N*** = 880 n (%)	Singleton ***N*** = 33,229 n (%)	Twin ***N*** = 794 n (%)	Singleton ***N*** = 31,524 n (%)	Twin ***N*** = 18,338 n (%)	Singleton ***N*** = 582,355 n (%)	
Groups compared and statistical significance reported^1^	A	A	B	B	C	C	
			D		D		
Hospital category for birth^ABCD^							
Public principal referral/women’s	573 (65)	8608 (26)	324 (41)	8204 (26)	8689 (47)	189,260 (32)	
Large public	141 (16)	9397 (28)	394 (50)	17,171 (54)	4595 (25)	209,070 (36)	
Medium/small public	105 (12)	12,032 (36)	42 (5)	4759 (15)	318 (2)	35,198 (6)	
Private	54 (6)	2730 (8)	28 (4)	1201 (4)	4658 (25)	146,078 (25)	
Born outside hospital	7 (1)	345 (1)	6 (1)	189 (1)	78 (< 1)	2749 (< 1)	
Mother transferred prior to birth^2,ABCd^	116 (13)	1358 (4)	40 (17)	559 (6)	616 (11)	3456 (2)	
Time from home to hospital (median (IQR))^ABCD^	38 (20, 401)	19 (9, 51)	21 (11, 49)	19 (10, 39)	20 (11, 35)	17 (11, 28)	
0–29 min	324 (37)	19,695 (60)	440 (56)	20,668 (66)	12,631 (69)	437,239 (76)	
30–59 min	146 (17)	3110 (9)	174 (22)	5833 (19)	3604 (20)	103,700 (18)	
1 h-2 h	80 (9)	2889 (9)	108 (14)	3522 (11)	1558 (9)	31,413 (5)	
3 h or greater	269 (31)	4342 (13)	66 (8)	1227 (4)	392 (2)	4529 (1)	
Location of WA private hospital unknown	54 (6)	2730 (8)	–	–	–	–	
Presentation^ABC^							
Vertex	558 (63)	31,693 (95)	196 (67)	11,957 (96)	4532 (73)	200,288 (96)	
Breech	291 (33)	1165 (4)	84 (29)	389 (3)	1482 (24)	7561 (4)	
Other	31 (4)	371 (1)	11 (4)	97 (1)	215 (3)	1581 (1)	
Delivery^ABCD^							
Vaginal birth	427 (49)	25,564 (77)	317 (40)	24,469 (78)	6765 (37)	421,801 (72)	
Spontaneous labour	*258 (29)*	*19,269 (58)*	*212 (27)*	*18,411 (58)*	*3706 (20)*	*302,880 (52)*	
Induced labour	*169 (19)*	*6295 (19)*	*105 (13)*	*6058 (19)*	*3059 (17)*	*118,921 (20)*	
Caesarean	453 (51)	7665 (23)	477 (60)	7022 (22)	11,567 (63)	160,129 (28)	
*Labour*							
*Spontaneous labour*	*134 (15)*	*2279* (7)	*187 (24)*	*2112* (7)	*3032 (17)*	*40,546 (7)*	
*Induced labour*	*39 (4)*	*1440* (4)	*16 (2)*	*1225* (4)	*849 (5)*	*27,946 (5)*	
*No labour*	*280 (32)*	*3946 (12)*	*274 (35)*	*3685 (12)*	*7686 (42)*	*91,637 (16)*	
*Planned/emergency*			–	–	–	–	
*Planned caesarean*	*166 (19)*	*2999* (9)	–	–	–	–	
*Emergency caesarean*	*287 (33)*	*4666 (14)*	–	–	–	–	
Instruments used^Cd^							
No	803 (91)	30,084 (91)	755 (95)	29,526 (94)	16,912 (92)	519,869 (89)	
Yes	77 (9)	3145 (9)	39 (5)	1973 (6)	1420 (8)	62,182 (11)	
Accoucheur (WA)^3^							Model of care (NSW)^3^
Obstetrician^A^	319 (36)	5683 (17)	42 (14)	1298 (10)	2818 (45)	75,804 (36)	Private obstetrician^bCD^
Other medical practitioner^A^	553 (63)	8842 (27)	223 (76)	5274 (42)	3274 (52)	77,227 (37)	Hospital-based medical^bCD^
Midwife^A^	195 (22)	19,998 (60)	30 (10)	2260 (18)	150 (2)	15,807 (8)	General practitioner^BCD^
Student^A^	82 (9)	4569 (14)	192 (65)	8948 (72)	3570 (57)	132,379 (63)	Hospital-based midwife^bCd^
Self/other/unknown	28 (3)	855 (3)	0 (0)	20 (< 1)	9 (< 1)	519 (< 1)	Independent midwife

*IQR* Interquartile range. ^1^The superscripts ^ABCDabcd^ which appear next to the demographic factors indicate whether two groups were statistically significantly different: ^A^ or ^a^ for a comparison of WA Aboriginal singletons and twins; ^B^ or ^b^ for a comparison of NSW Aboriginal singletons and twins; ^C^ or ^c^ for a comparison of NSW non-Aboriginal singletons and twins; and ^D^ or ^d^ for a comparison of NSW Aboriginal twins and NSW non-Aboriginal twins. Pearson’s chi-squared tests were conducted. A capital letter indicates *p* < 0.001 and a lower-case letter indicates 0.001 < *p* < 0.05. ^2^Mother transferred prior to birth was only collected comprehensively from 2007 in NSW so only data from 2007 and 2008 births are reported. ^3^Categories of accoucheur (WA) and model of care (NSW) are not mutually exclusive. Data were missing for: hospital category for birth (117 infants in WA, 0 in NSW), mother transferred prior to the birth (434 infants in WA, 440817 in NSW), time from home to hospital (469 infants in WA, 5907 in NSW), presentation (0 infants in WA, 404617 in NSW), delivery (0 infants in WA, 464 in NSW), instruments used (0 infants in WA, 335 in NSW), and model of care (404,184 in NSW)

Mothers of twins were more likely to be transferred to another hospital prior to the birth (WA Aboriginal twins and singletons: 13 and 4%, NSW Aboriginal twins and singletons: 17 and 6%, and NSW non-Aboriginal twins and singletons: 11 and 2%). Mothers of Aboriginal twins often gave birth far from home. In WA, almost a third (31%) of the Aboriginal twin births in hospital were at least 3 h by road from the mother’s home; in NSW, the percentage was 8%.

Caesarean section was the most common method of birth for twins in all three groups and this was more than twice as common for twins than singletons. NSW non-Aboriginal twins were the most likely to be born by caesarean section (63%), followed by NSW Aboriginal twins (60%) and WA Aboriginal twins (51%). 63% (287/453) of WA Aboriginal twin caesarean sections were categorised as emergency caesarean sections.

In WA, twin births were twice as likely to be attended by obstetricians (36%) as singleton births (17%). In NSW, Aboriginal twins were much more likely to have a hospital-based medical model of care (76%) than Aboriginal singletons (42%). Private obstetricians were involved in a large proportion of non-Aboriginal NSW twin births (45%), compared to only 14% for Aboriginal twin births.

### Perinatal outcomes

Twins were only slightly more likely than singletons to be stillborn, but in both states 3% of liveborn Aboriginal twins died within 28 days, compared with less than half a percent of Aboriginal singletons (Table 4). Twins were roughly one-kilogram lighter at birth than singletons for both Aboriginal and non-Aboriginal liveborn infants. Aboriginal twins from WA had the lowest mean weight at 2225 g. Among liveborn infants, 35% of WA Aboriginal twins were born at 37 or more weeks’ gestational age compared to 42% of NSW Aboriginal twins and 51% of NSW non-Aboriginal twins. Twins were more than twice as likely to be admitted to a NICU/SCN and twin infants and their mothers both averaged at least twice as long in hospital as singleton infants and their mothers. The median length of stay in hospital was 7 days for WA Aboriginal twins and NSW Aboriginal 2009/2012 school starters twins (interquartile range [IQR]: 4–14 days for WA Aboriginal twins and 4–16 days for NSW Aboriginal twin 2009/2012 school starters) and 6 days [IQR: 4–8 days] for their mothers (Table 4 and Additional file 2, Table S1). Selected perinatal outcomes for triplets are reported in Additional file 1.

**Table 4:** Perinatal outcomes in Western Australia (2000–2013) and New South Wales (2002–2008) by plurality and Aboriginality

	Western Australia (2000–2013)		New South Wales (2002–2008)			
	Aboriginal		Aboriginal		Non-Aboriginal	
Perinatal outcomes	Twin ***N*** = 880 n (%)	Singleton ***N*** = 33,229 n (%)	Twin ***N*** = 794 n (%)	Singleton ***N*** = 31,524 n (%)	Twin ***N*** = 18,338 n (%)	Singleton ***N*** = 582,355 n (%)
Groups compared and statistical significance reported^1^	A	A	B	B	C	C
			D		D	
**All births**	***N*** **= 880**	***N*** **= 33,229**	***N*** **= 794**	***N*** **= 31,524**	***N*** **= 18,338**	***N*** **= 582,355**
Stillbirth^aC^	20 (2)	398 (1)	10 (1)	262 (1)	332 (2)	3295 (1)
**Live births only**	***N*** **= 860**	***N*** **= 32,831**	***N*** **= 784**	***N*** **= 31,262**	***N*** **= 18,006**	***N*** **= 579,060**
Neonatal death^ABC^	29 (3)	147 (< 1)	20 (3)	105 (< 1)	294 (2)	1182 (< 1)
Birth weight (grams) (mean (SD))	2225 (682)	3207 (636)	2311 (661)	3283 (622)	2436 (605)	3419 (545)
Birth weight bands ^ABCD^						
< 1000	59 (7)	252 (1)	35 (4)	174 (1)	535 (3)	1673 (< 1)
1000–1499	67 (8)	315 (1)	67 (9)	224 (1)	840 (5)	2070 (< 1)
1500–1999	153 (18)	709 (2)	101 (13)	549 (2)	2213 (12)	4458 (1)
2000–2499	240 (28)	2292 (7)	239 (31)	1895 (6)	5101 (28)	16,228 (3)
2500–2999	248 (29)	7097 (22)	251 (32)	5970 (19)	6477 (36)	81,429 (14)
3000–3499	88 (10)	11,544 (35)	72 (9)	10,742 (34)	2498 (14)	212,821 (37)
3500+	5 (1)	10,622 (32)	18 (2)	11,695 (37)	320 (2)	260,131 (45)
Gestational age (median (IQR))	36 (33, 37)	39 (38, 40)	36 (34, 37)	39 (38, 40)	37 (35, 38)	39 (38, 40)
Gestational age categories ^ABCD^						
20–23	22 (3)	62 (< 1)	9 (1)	52 (< 1)	152 (1)	479 (< 1)
24–27	30 (3)	183 (1)	24 (3)	139 (< 1)	345 (2)	1200 (< 1)
28–31	68 (8)	414 (1)	75 (10)	261 (1)	1001 (6)	2712 (< 1)
32–34	198 (23)	922 (3)	126 (16)	743 (2)	2658 (15)	7137 (1)
35–36	240 (28)	2329 (7)	211 (27)	1638 (5)	4475 (25)	18,783 (3)
37–38	279 (32)	10,030 (31)	292 (38)	7119 (23)	8216 (46)	124,134 (21)
39+	23 (3)	18,846 (57)	31 (4)	21,298 (68)	861 (5)	424,508 (73)
Resuscitation^ABC^	449 (52)	11,170 (34)	455 (58)	13,190 (42)	10,463 (58)	235,578 (41)
Length of stay (median (IQR) in days)^A^	7 (4–14)	3 (2–4)	–	–	–	–
Admitted to NICU or SCN^2,ABCd^	452 (64)	4343 (29)	500 (64)	5984 (19)	10,521 (57)	82,257 (14)
Cerebral palsy^.^	5 (1)	114 (< 1)	–	–	–	–
Respiratory distress syndrome of newborns^A^	146 (17)	883 (3)	–	–	–	–
**Maternal outcomes**						
PPH requiring blood transfusion^AC^	36 (4)	607 (2)	10 (3)	403 (3)	175 (3)	3389 (2)
Mother’s length of stay (median (IQR) in days)^A^	6 (4–8)	3 (2–5)	–	–	–	–

*SD* standard deviation, *IQR* Interquartile range. ^1^The superscripts ^ABCDabcd^ which appear next to the demographic factors indicate whether two groups were statistically significantly different: ^A^ or ^a^ for a comparison of WA Aboriginal singletons and twins; ^B^ or ^b^ for a comparison of NSW Aboriginal singletons and twins; ^C^ or ^c^ for a comparison of NSW non-Aboriginal singletons and twins; and ^D^ or ^d^ for a comparison of NSW Aboriginal twins and NSW non-Aboriginal twins. Pearson’s chi-squared tests were conducted for all variables except length of stay. Wilcoxon rank sum tests were conducted for infant and maternal length of stay. A capital letter indicates *p* < 0.001 and a lower-case letter indicates a 0.001 < *p* < 0.05. ^2^In WA, admission to a NICU or SCN in the hospital the infant was born in and admission to a NICU in any hospital they were transferred to were included (time in a SCN after being transferred to another hospital was not available). In NSW, admission to a NICU or SCN in the hospital the infant was born in was included (admission to a NICU or SCN after transfer was not available). Data were missing for: birth weight (0 infants in WA, 286 in NSW), gestational age (45 infants in WA, 433 in NSW), resuscitation (0 infants in WA, 932 in NSW), admitted to NICU or SCN (18,122 infants in WA, 581 in NSW), PPH requiring blood transfusion (0 infants in WA, 401967 in NSW) and mother’s length of stay (90 cases in WA). No data were missing for stillbirth, neonatal death, infant length of stay, cerebral palsy or respiratory distress syndrome of newborns

## Discussion

This large population-based study in two Australian states showed that the journey from pregnancy to discharge from hospital presented major challenges for many Aboriginal mothers and their twins. Pregnancy complications, birth interventions, travel far from home and adverse perinatal outcomes were more common for Aboriginal twins than Aboriginal singletons, consistent with data for non-Aboriginal singletons and twins, and more common for Aboriginal twins than non-Aboriginal twins.

As expected, mothers of twins in the Aboriginal and non-Aboriginal cohorts had higher proportions of pregnancy complications than singletons, except for gestational diabetes among non-Aboriginal births. The time period covered by this study largely preceded changes in the diagnostic criteria and testing for gestational diabetes which were progressively implemented in Australian jurisdictions following the publication of the Hyperglycemia and Adverse Pregnancy Outcome (HAPO) study in 2008 [28]. These changes greatly increased diagnoses of gestational diabetes [28] so the proportion of women diagnosed with gestational diabetes in this study (approximately 5%) is low compared to the national percent of 13.5% reported recently [1]. Our finding of similar prevalence of gestational diabetes among twin and singleton births in both WA and NSW Aboriginal births and only a slight difference for NSW non-Aboriginal births is consistent with some other studies [29, 30], though not all [7, 31, 32]. The slight increase in gestational diabetes for NSW non-Aboriginal mothers of twins may be due to maternal age as gestational diabetes is more common among older women [33] and Egeland and Irgens found that the higher risk for multiple pregnancies was largely attenuated after adjustment for maternal age [32]. It is also possible that some diagnoses are missed for Aboriginal women; a study in remote WA of births in 2013 found that Aboriginal women were less likely to be screened for gestational diabetes with the recommended oral glucose tolerance test [34]. Hypertensive disorders, including pre-eclampsia, eclampsia and gestational hypertension combined, were over twice as common in twin pregnancies, compared to singletons, as found in other populations [35–37]. Prior twin cohort studies have found that hypertensive disorders often onset earlier and have greater severity in twin pregnancies compared to singletons [37, 38].

While pregnancy complications were more common among twin pregnancies compared with singleton pregnancies, percentages were generally similar for NSW Aboriginal and non-Aboriginal twin pregnancies. However, there was some evidence that gestational diabetes and hypertensive disorders were more common in mothers of non-Aboriginal twins and antepartum haemorrhage was roughly twice as common for mothers of Aboriginal twins (13%) than non-Aboriginal twins (6%) in NSW 2009/2012 school starters. This was noteworthy as rates were similar for Aboriginal and non-Aboriginal singleton pregnancies (4 and 3%, respectively, Table S1) and those singleton rates were in line with findings from other Australian jurisdictions [39]. Additionally, another Australian study on (predominantly non-Aboriginal) twin pregnancies found antepartum haemorrhage affected only 5% of pregnancies [40]. Possible explanations include that this was a chance result in this subsample of NSW Aboriginal twins or that antepartum haemorrhage risk factors which are more common among Aboriginal mothers (for example, increased parity and tobacco use [21]) pose a particularly high risk for twin pregnancies, and further investigation is warranted.

We found that Aboriginal twins had more adverse perinatal outcomes than Aboriginal singletons and non-Aboriginal NSW twins. Perinatal death (stillbirth or neonatal death) was higher for twins (compared to singletons) in both states, particularly WA Aboriginal twins. The persistent gap between Aboriginal and non-Aboriginal singleton birth weight and gestational age was also apparent among twins [1]. There was marked a difference between the proportion of Aboriginal and non-Aboriginal twins born at 37 weeks’ gestation or more (35% of WA Aboriginal, 42% of NSW Aboriginal and 51% of NSW non-Aboriginal twins), despite similar prevalence of pregnancy complications and a greater proportion of mothers of Aboriginal twins aged 20 to 34 years, an age range with lower risk of preterm birth at the population level [41, 42]. The increased risk of preterm birth for Aboriginal twins may result from factors associated with spontaneous preterm birth which were not explored in this study, such as infection and smoking [43, 44]. Another explanation may be that although Aboriginal twins were more likely to be born in facilities with greater medical oversight than Aboriginal singletons (a higher percentage of twin births were at principal referral or specialist women’s hospitals and obstetricians were more likely to be present), their health care still differed from non-Aboriginal twins.

This study is the first to describe in detail the pregnancies and births of Aboriginal twins, providing baseline data to inform the response of health services to this important population. Many studies exclude multiples from analyses or provide limited information about Aboriginal twins. Our study covered almost half of the Aboriginal Australian population [20] ensuring sufficient twins for meaningful estimates in each state and allowing assessment of whether patterns were consistent in both states. While these communities share some common recent history, including colonisation and its effects, major differences exist within, and between, these states, including cultural and linguistic differences, remoteness and availability of healthcare. Both have large urban populations (40% of Aboriginal people in WA and 46% in NSW), but in WA, 24% of Aboriginal people live in very remote areas, compared to only 1% in NSW [20].

By using population-based linked data, selection bias – common in cohort studies or surveys using primary data collection – was minimised. Limitations of our study include that the data were collected for administrative, not research, purposes and lacked some relevant information such as reasons for giving birth in a distant hospital and the use of assisted reproductive technologies. However, Aboriginal women are much less likely to access assisted reproductive technologies than non-Aboriginal women – a study of 5 Australian states found that only 3% (9/304) of Aboriginal twin pregnancies were the result of assisted reproductive technology, compared to 24% (2280/9519) of non-Aboriginal mothers [40]. There were also differences in information recorded in WA and NSW, though most data were comparable. Additionally, different methods of identifying Aboriginal infants were used in the two states and some data could only be reported for a subset of NSW births because linked hospital data were not available for all NSW births. While approximate travel times were an indicator of distance from home and community at the time of birth, the mother’s actual method of transport to hospital was not recorded. Finally, mothers from border regions may have travelled interstate, as is common practice in the Far West Local Health District of NSW. As such, some information on birth outcomes may be missing from our data and may disproportionately include high-risk multiple births among Aboriginal women.

Our findings highlight that it is more common for the mothers of Aboriginal twins to face a number of practical challenges that may impact on their antenatal care, pregnancy and birth outcomes than mothers of Aboriginal singletons and non-Aboriginal twins; challenges that have major implications for birth planning and the birth experience for mothers of Aboriginal twins. Specialised medical care and NICUs are concentrated in large urban areas and many mothers needed to travel to access that care. We did not have data about the location of antenatal scans and appointments, but travel may also have been necessary during high-risk twin pregnancies. Mothers may have missed valuable support from their family and community for extended periods of time. This travel may also have led to substantial costs for additional antenatal scans, transport, accommodation and, potentially, childcare for older children and reduced work-related income for herself and any accompanying support people. At present, some government support is available for women to travel. For example, NSW women who must travel over 100 km can access the Isolated Patients Travel and Accommodation Assistance Scheme, although the full costs of travel and accommodation are not covered and food is not subsidised [45].

While all mothers of twins face challenges, the circumstances of many Aboriginal women mean that these challenges may be more difficult to overcome. Aboriginal women report loneliness and distress with relocation for birth [46]. Additionally, costs associated with antenatal care and birth may impact on Aboriginal women who are disproportionately socio-economically disadvantaged following over 200 years of colonisation. Aboriginal women are also more likely to have older children (only 27 and 28% of mothers of Aboriginal twins in WA and NSW were nulliparous compared to 44% of mothers of non-Aboriginal twins) and these children may need to travel with their mothers or be cared for by others. Some women may be distressed that their babies are not born on country [46]. Finally, Aboriginal women often access antenatal and postnatal care through primary health care organisations operated by local Aboriginal communities. Births in a distant hospital may require a transfer of care from midwives at these local Aboriginal services to a large, mainstream hospital which may be perceived as less culturally safe and disrupt continuity of care [47].

Given the range of challenges that mothers of twins may face during pregnancy and birth, this group needs more support than mothers of singletons. While Australian State and Federal Governments currently fund services and develop guidelines for specific groups of mothers (for example, teenage mothers, nulliparous mothers, Aboriginal mothers), as far as we are aware, there are no targeted supports for parents of twins, let alone mothers of Aboriginal twins who are more likely to live in remote and socioeconomically deprived areas, be younger, and have other children than mothers of non-Aboriginal twins. Aboriginal mothers of twins are a small group, but they are an important high-risk group, and these mothers should be considered for targeted support. Research with parents and Aboriginal community controlled health organisations (particularly in remote areas) and the major hospitals where many Aboriginal twins are born is needed to identify upfront costs during pregnancy for the birth and the impact of these costs, any medical or psychosocial consequences of relocation for birth, whether additional financial or other practical support is needed and, if so, the optimal timing of the support.

## Conclusions

Many Aboriginal twin pregnancies and births are physically and practically challenging and the majority of multiples are born early and small. This study highlights the importance of policies that support health services to meet the practical, financial and psychosocial needs of mothers and families, in addition to meeting their health needs.

## Supplementary Information

**Additional file 1.** Additional methodological details.

**Additional file 2: Table S1.** Characteristics and outcomes of mothers and their singleton and twin infants born in Western Australia from 2000 to 2013 and the New South Wales 2009/2012 school starters by plurality and Aboriginality.

**Additional file 3: Table S2.** Codes of the International Classification of Diseases, 10th Revision (ICD-10-AM) and Australian Classification of Health Interventions (ACHI) used to identify maternal health conditions and complications during pregnancy from hospital records and Midwives Notification System (MNS, WA) data and Perinatal Data Collection (PDC, NSW).

## Data Availability

The data that support the findings of this study are available from the WA and NSW government agencies, but restrictions apply to the availability of these data, which were used under license for the current study, and so are not publicly available. Data are however available with relevant ethics permissions and approval of the data custodians of each of the datasets used in this study. For information about data access, approval from the data custodians and data linkage, researchers can contact the WA Data Linkage Branch (https://www.datalinkage-wa.org.au/) regarding the Defying the Odds study data and the NSW Centre for Health Record Linkage (http://www.cherel.org.au/) regarding the Seeding Success study data.

## Abbreviations

AEDC: Australian Early Development Census (AEDC)
ICD-10-AM: International Statistical Classification of Diseases, Tenth Revision, Australian Modification
IQR: Interquartile range
NICU: Neonatal intensive care unit
NSW: New South Wales
PPH: Post-partum haemorrhage
SCN: Special care nursery
WA: Western Australia
