# Supplementary material for: Pregnancy and birth characteristics of Aboriginal twins in two Australian states: a data linkage study
Source: BMC Pregnancy Childbirth. 2021 Jun 28;21:448. doi: 10.1186/s12884-021-03945-9 (PMC8240393; doi:10.1186/s12884-021-03945-9)
Supplement: Supplementary file 1 — Additional file 1. Additional methodological details. [file 12884_2021_3945_MOESM1_ESM.docx]

**Additional file 1: Pregnancy and birth characteristics of Aboriginal twins in two Australian states: a data linkage study**

**1. Linkage of multiples**

In the WA study population, all infants were linked to their mothers and twins and higher order multiples were linked to each other based on their mother’s unique identifier, their year of birth and plurality. One twin did not link to their corresponding twin and was excluded.

In the NSW study population, 19729 multiples needed to be linked – the 19706 multiples in the final sample and 23 that were excluded. Only 7188 (36%) of these children were linked to their mothers as part of the Seeding Success study^1^ and they could be linked to each other in the same way as the WA multiples.

The remaining multiples were initially linked to each other if they had the same month of birth, maternal month of birth, Statistical Local Area of residence and plurality. In a small proportion of cases (14), this information was not specific enough and gestational age and the hospital where they were born were also used. Following this process, 59 multiples had not linked. The requirement that they had the same place of residence recorded on their birth record was then dropped and 28 then linked. We then removed the requirement of the same month of birth for the remaining 31 multiples (some twins in the data were born on different days) and 14 more linked. The final 17 multiples that still did not link to their twins or triplets were excluded. It is possible some were singletons incorrectly recorded as twins or triplets.

Manual checks were then conducted of cases where twin pairs had a significant amount of inconsistent information for a range of variables including gestational age, parity, mother’s country of birth, and maternal health conditions. Three linkages were considered unsafe and those three twin pairs were also excluded.

As a sensitivity analysis, an alternate method of linkage was undertaken, with very similar results. This matching included additional information about the mother. Initial matching was on infant and maternal month of birth, plurality, antenatal care, Statistical Local Area of residence, maternal Aboriginality and maternal country of birth. The variables for linking those tuples who still did not link were then reduced to maternal variables only – maternal month of birth, Statistical Local Area of residence, Aboriginality and country of birth. This method of matching resulted in two additional non-Aboriginal twin pairs.

**2. Socioeconomic disadvantage**

Quintiles of socio-economic disadvantage were formed using the Australian Bureau of Statistics’ Socio-Economic Indexes for Areas (SEIFA) Index of Relative Socio-Economic Disadvantage (IRSD).^2^ The IRSD score for a geographical region is based on the socio-economic status of the residents of the region, calculated using their responses to the Australian Census. For the WA cohort, we used 2011 IRSD scores for the mother’s Statistical Area 1 (SA1).^2^ For the NSW cohort, 2006 IRSD scores were based on the mother’s Statistical Local Area (SLA) of residence at birth. Quintiles of socioeconomic deprivation were formed using ABS cut points of IRSD scores that divide Australian SA1s and SLAs into five equal groups.^2^

**3. Travel time to hospital by road**

The travel time by road from the mother’s place of residence to the hospital where she gave birth was estimated. The start of the road route was the centroid of the geographic unit which contained the mother’s residential address and the end of the route was the hospital where the birth took place.

*Start of road route: mother’s place of residence*

The starting point was the centroid of the geographical unit where the mother resided. R software, package *rgdal*,^3^ was used to find the latitude and longitude coordinates for each centroid from ABS shapefiles available online.^4, 5^ A small number of centroids were far from roads and travel times could not be calculated (13 centroids in WA and 1 in NSW) so the starting positions were manually changed to the nearest recognisable road or town to create a feasible route.

The smallest geographic unit available in the data for each mother’s place of residence was used to minimise imprecision. For the WA data, SA1 2011 was used. They had a median area of 0.25 km^2^ in 2011, 92% were less than 10 km^2^ and 95% were less than 100 km^2^.^2, 4^ However, some SA1s are very large in remote areas of WA – 8 of the 5,508 SA1s covered over 100,000 km^2^. Travel times could not be estimated for 2784 births (8%) in a private hospital as private hospitals were not individually identified in the data. Travel times could not be estimated for a further 469 births (1%) as the infants were born outside hospital (352 births) or the birth could not be linked to a hospital record (117 births).

For NSW the smallest geographic unit available was SLA 2006 in the Perinatal Data Collection. SLAs are larger than SA1s and therefore estimates of travel time for NSW are less precise than estimates for WA. Travel times could not be assigned for 5907 (1%) births of singletons and twins as the mother’s SLA was not listed. The majority of these mothers were recorded as interstate or overseas residents.

*End of road route: hospital*

The latitude and longitude coordinates of the hospitals were obtained from the MyHospital, Australian Institute of Health and Welfare website.^6^ Co-ordinates for eight WA hospitals and six NSW hospitals were not available from MyHospital due to hospital location changes and closures during the study period and they were obtained from Google Maps.

*Road travel times*

The travel times were calculated using the statistical software STATA with the *georoute* package ^7, 8^.

In NSW, births outside hospital under the care of an independent midwife could be identified and travel time was set to 0 minutes. In WA, home births could not be distinguished from all other births outside hospital, such as births *en route* to hospital, and all births recorded as ‘born before arrival’ in the Midwives Notification System were set to missing. Women living on Lord Howe Island and Rottnest Island who gave birth at a mainland hospital were assumed to have travelled more than 3 hours.

**4. Perinatal outcomes of triplets**

Triplets were born much lighter and earlier than twins. In WA, the average birth weight was 1201g (standard deviation (sd) 818g) and median gestational age of 27 weeks (interquartile range (IQR) 23-34 weeks). Aboriginal and non-Aboriginal triplets in NSW averaged 1609g (sd 733g) and 1657g (sd 522g), respectively, and their median gestational age was 32 weeks (IQR Aboriginal 29-35; IQR non-Aboriginal 30-34).

**References**

1. Falster K, Jorgensen M, Hanly M, Banks E, Brownell M, Eades S, et al. Data Resource Profile: Seeding Success: a cross-sectoral data resource for early childhood health and development research in Australian Aboriginal and non-Aboriginal children. Int J Epidemiol. 2017;46(5):1365-6j.

2. Australian Bureau of Statistics. Census of Population and Housing: Socio-Economic Indexes for Areas (SEIFA), Australia - Data only , 2006. Cat. No. 2033.0.55.001 [Internet]. Canberra: Australia Bureau of Statistics; 2008. https://www.abs.gov.au/AUSSTATS/abs@.nsf/DetailsPage/2033.0.55.0012006?OpenDocument. Accessed 13 May 2020.

3. Bivand R, Keitt T, Rowlingson B. rgdal: Bindings for the 'Geospatial' Data Abstraction Library. R package version 1.4-2. 2019. https://CRAN.R-project.org/package=rgdal.

4. Australian Bureau of Statistics. Australian Statistical Geography Standard (ASGS): Volume 1 - Main Structure and Greater Capital City Statistical Areas, July 2011. Cat. No. 1270.0.55.001. 2011. https://www.abs.gov.au/AUSSTATS/abs@.nsf/DetailsPage/1270.0.55.001July%202011?OpenDocument. Accessed 22 Aug 2019.

5. Australian Bureau of Statistics. Australian Standard Geographical Classification (ASGC), Digital Boundaries, 2006. Cat. No. 1259.0.30.002. https://www.abs.gov.au/AUSSTATS/abs@.nsf/DetailsPage/1259.0.30.0022006. Accessed 24 Jul 2020.

6. Australian Institute of Health and Welfare. Hospital contact details. Canberra: AIHW; 2019. https://www.myhospitals.gov.au/about-the-data/download-data. Accessed 19 Sep 2019.

7. Weber S, Péclat M. A simple command to calculate travel distance and travel time. The Stata Journal. 2017;17(4):962-71.

8. StataCorp. Stata Statistical Software: Release 15. College Station, TX: StataCorp LLC2017.
