# Supplementary material for: Pregnancy and birth characteristics of Aboriginal twins in two Australian states: a data linkage study
Source: BMC Pregnancy Childbirth. 2021 Jun 28;21:448. doi: 10.1186/s12884-021-03945-9 (PMC8240393; doi:10.1186/s12884-021-03945-9)
Supplement: Supplementary file 2 — Additional file 2: Table S1. Characteristics and outcomes of mothers and their singleton and twin infants born in Western Australia from 2000 to 2013 and the New South Wales 2009/2012 school starters by plurality and Aboriginality. [file 12884_2021_3945_MOESM2_ESM.docx]

**Additional file 2: Pregnancy and birth characteristics of Aboriginal twins in two Australian states: a data linkage study**

**Table S1: Characteristics and outcomes of mothers and their singleton and twin infants born in Western Australia from 2000-2013 and the New South Wales 2009/2012 school starters by plurality and Aboriginality**

|  | **Western Australia  (born 2000-2013)** | |  | **New South Wales  (2009/2012 school starters)** | | | |
| --- | --- | --- | --- | --- | --- | --- | --- |
|  | **Aboriginal** | |  | **Aboriginal** | | **Non-Aboriginal** | |
| **Characteristics and outcomes** | **Twin N=880 n (%)** | **Singleton  N=33,229 n (%)** |  | **Twin N=199 n (%)** | **Singleton  N=8,275 n (%)** | **Twin N=4,562 n (%)** | **Singleton  N=151,552 n (%)** |
| Groups compared and statistical significance reported^1^ | A | A |  | B | B | C | C |
|  |  |  |  | D |  | D |  |
|  |  |  |  |  |  |  |  |
| **Demographics** |  |  |  |  |  |  |  |
| Insurance at time of birth^abCd^ | 64 (7) | 1621 (5) |  | 26 (13) | 545 (7) | 1930 (44) | 53108 (36) |
|  |  |  |  |  |  |  |  |
| **Pregnancy** |  |  |  |  |  |  |  |
| Maternal health and pregnancy complications |  |  |  |  |  |  |  |
| Gestational diabetes^C^ | 48 (5) | 1724 (5) |  | 12 (6) | 368 (4) | 347 (8) | 8517 (6) |
| Pre-existing diabetes^C^ | 8 (1) | 557 (2) |  | n.p. | 66 (1) | 20 (<0) | 770 (1) |
| Pre-eclampsia/eclampsia/gestational hypertension^ABC^ | 148 (17) | 2650 (8) |  | 40 (20) | 669 (8) | 808 (18) | 12218 (8) |
| Pre-existing hypertension^C^ | 18 (2) | 415 (1) |  | 5 (3) | 98 (1) | 85 (2) | 1855 (1) |
| Threatened abortion^AC^ | 56 (6) | 1002 (3) |  | 5 (3) | 68 (1) | 48 (1) | 674 (<1) |
| Antepartum haemorrhage^ABCD^ | 84 (10) | 1787 (5) |  | 25 (13) | 333 (4) | 252 (6) | 5014 (3) |
| Threatened preterm labour^ABC^ | 256 (29) | 3099 (9) |  | 31 (16) | 436 (6) | 606 (13) | 4025 (3) |
| Preterm prelabour rupture of membranes^ABC^ | 174 (20) | 1695 (5) |  | 18 (9) | 201 (3) | 544 (12) | 2592 (2) |
|  |  |  |  |  |  |  |  |
| **Outcomes** |  |  |  |  |  |  |  |
| *Infant outcomes* |  |  |  |  |  |  |  |
| Length of stay (median (IQR) in days)^ABC^ | 7 (4-14) | 3 (2-4) |  | 7 (4-16) | 3 (2-4) | 7 (5-16) | 3 (2-5) |
| Admitted to NICU or SCN^2,ABC^ | 452 (64) | 4343 (29) |  | 130 (65) | 1605 (19) | 2708 (59) | 22130 (15) |
| Respiratory distress syndrome of newborns^ABC^ | 146 (17) | 883 (3) |  | 21 (11) | 171 (2) | 456 (10) | 2074 (1) |
| *Maternal outcomes* |  |  |  |  |  |  |  |
| PPH requiring blood transfusion^AbCd^ | 36 (4) | 607 (2) |  | 11 (6) | 232 (3) | 125 (3) | 2214 (1) |
| Mother's length of stay (median (IQR) in days)^ABC^ | 6 (4-8) | 3 (2-5) |  | 6 (4-8) | 3 (2-5) | 6 (5-8) | 4 (3-5) |

IQR: Interquartile range. ^1^The superscripts ^ABCDabcd^ which appear next to the demographic factors indicate whether two groups were statistically significantly different: ^A^ or ^a^ for a comparison of WA Aboriginal singletons and twins; ^B^ or ^b^ for a comparison of NSW Aboriginal singletons and twins; ^C^ or ^c^ for a comparison of NSW non-Aboriginal singletons and twins; and ^D^ or ^d^ for a comparison of NSW Aboriginal twins and NSW non-Aboriginal twins. Pearson's chi-squared tests were conducted for all variables except length of stay. Wilcoxon rank sum tests were conducted for infant and maternal length of stay. A capital letter indicates p < 0.001 and a lower case letter indicates a 0.001 < p < 0.05. ^2^Admission to a NICU or SCN in the hospital the infant was born in and admission to a NICU in any hospital they were transferred to were included (time in a SCN after being transferred to another hospital was not available). Data were missing for: insurance at the time of birth (380 infants in WA, 5668 infants in NSW), threatened abortion, antepartum haemorrhage, threatened preterm labour and preterm prelabour rupture of membranes (0 infants in WA, 2835 infants in NSW, for each condition), infant's length of stay (0 infants in WA, 1273 infants in NSW), respiratory distress syndrome of newborns (0 infants in WA, 1273 infants in NSW), and mother's length of stay (90 infants in WA, 2945 infants in NSW). No data were missing for gestational or pre-existing diabetes, pre-eclampsia/eclampsia/gestational hypertension, pre-existing hypertension, admitted to NICU or SCN and PPH requiring blood transfusion. For some NSW twin pairs, only one of the two children started school in 2009/2012 and had an Australian Early Development Census record.
