# Supplementary material for: Pregnancy and birth characteristics of Aboriginal twins in two Australian states: a data linkage study
Source: BMC Pregnancy Childbirth. 2021 Jun 28;21:448. doi: 10.1186/s12884-021-03945-9 (PMC8240393; doi:10.1186/s12884-021-03945-9)
Supplement: Supplementary file 3 — Additional file 3: Table S2. Codes of the International Classification of Diseases, 10th Revision (ICD-10-AM) and Australian Classification of Health Interventions (ACHI) used to identify maternal health conditions and complications during pregnancy from hospital records and Midwives Notification System (MNS, WA) data and Perinatal Data Collection (PDC, NSW). [file 12884_2021_3945_MOESM3_ESM.docx]

**Additional file 3: Pregnancy and birth characteristics of Aboriginal twins in two Australian states: a data linkage study**

**Table S2: Codes of the International Classification of Diseases, 10^th^ Revision (ICD-10-AM) and Australian Classification of Health Interventions (ACHI) used to identify maternal health conditions and complications during pregnancy from hospital records and Midwives Notification System (MNS, WA) data and Perinatal Data Collection (PDC, NSW)**

| **Diagnosis / procedure** | **ICD-10-AM codes in hospital records** | **Collected in MNS/PDC during study period** | |
| --- | --- | --- | --- |
| Gestational diabetes | O24.4, O24.9 | MNS, PDC | |
| Pre-existing diabetes | E10-E14, O24.0-O24.3, O24.5 | MNS, PDC | |
| Pre-eclampsia/eclampsia/gestational hypertension | O13, O14, O15, O16 | MNS, PDC | |
| Pre-existing hypertension | O10, O11 | MNS, PDC | |
| Threatened abortion | O20.0 (alongside a diagnosis of O09.0-O09.2 or estimated gestational age less than 20 weeks) | MNS (specified as prior to 20 weeks) | |
|  |  |  | |
| Antepartum haemorrhage | 044.1, O45, O46 | MNS | |
| Threatened preterm labour | O47.0 | MNS | |
| Premature rupture of membranes | O42 | MNS | |
| Preterm premature rupture of membranes | O42 (alongside a diagnosis of gestation less than 37 weeks [O09.0-O09.5] or estimated gestational age at date of admission less than 37 weeks^1^) | No (Premature rupture of membranes information in MNS was combined with estimated gestational age at time of admission less than 37 weeks) | |
| Respiratory distress of newborn | P22.0 | No | |
| Postpartum haemorrhage | O72 | MNS | |
| Postpartum haemorrhage with blood transfusion | O72 (with record of blood transfusion) | PDC | |
|  |  |  | |
|  | **ACHI codes in hospital records** |  | |
| Blood transfusion | 13706–01, 13706–02 | - | |
| ^1^ Gestational age at date of admission was estimated by subtracting the difference (in weeks) between the infant’s date of birth and their mother’s date of admission from using the infant’s estimated gestational age, as recorded on their birth record | | |  |

If a mother had records of both gestational and pre-existing diabetes for one birth, only one diagnosis was included. Diagnoses in the hospital record were prioritised over the birth record (Midwives Notification System in WA and Perinatal Data Collection in NSW) as a NSW study found the hospital record to be more reliable^1^ and a diagnosis of pre-existing diabetes was prioritised over a diagnosis of gestational diabetes, an approach used by the Australian Institute of Health and Welfare.^2^

**References**

1. Lain SJ, Hadfield RM, Raynes-Greenow CH, Ford JB, Mealing NM, Algert CS, et al. Quality of data in perinatal population health databases: a systematic review. Med Care. 2012;50(4):e7-20.

2. Australian Institute of Health and Welfare. Incidence of gestational diabetes in Australia: Methods. Canberra: AIHW; 2019. https://www.aihw.gov.au/reports/diabetes/incidence-of-gestational-diabetes-in-australia/contents/methods. Accessed 4 Jan 2020.
